# Biotechnological 2-Phenylethanol Production: Recent Developments

**DOI:** 10.3390/molecules29235761

**Published:** 2024-12-05

**Authors:** Ana R. S. Bernardino, Cristiana A. V. Torres, João G. Crespo, Maria A. M. Reis

**Affiliations:** 1 Laboratory i4HB, Institute for Health and Bioeconomy, NOVA School of Science and Technology, Universidade NOVA de Lisboa, 2829-516 Caparica, Portugal; ar.bernardino@campus.fct.unl.pt (A.R.S.B.); amr@fct.unl.pt (M.A.M.R.); 2 UCIBIO—Applied Molecular Biosciences Unit, Department of Chemistry, NOVA School of Science and Technology, Universidade NOVA de Lisboa, 2829-516 Caparica, Portugal; 3 LAQVREQUIMTE, Chemistry Department, NOVA School of Science and Technology, Universidade NOVA de Lisboa, 2829-516 Caparica, Portugal; jgc@fct.unl.pt; 4 ITQB, Universidade NOVA de Lisboa, Av. da República, 2780-157 Oeiras, Portugal

**Keywords:** 2-phenylethanol, bioproduction, microbial fermentation, in situ product removal techniques, low-cost feedstocks

## Abstract

2-Phenylethanol (2-PE) is a key flavor compound with a rose-like scent, used in the cosmetics, perfume, home care and food industries. This aroma compound can be obtained naturally from various flowers, however chemical synthesis is the most used route to meet market demand. The increasing interest in natural products has led to the development of more environmentally friendly alternatives for 2-PE production through biotechnological approaches. The most efficient approach involves the biotransformation of L-phenylalanine into 2-PE via the Ehrlich pathway, a process observed in different microorganisms such as yeasts and bacteria. 2-PE produced by this way can be considered as natural. However, due to the toxicity of the aroma to the producing microorganism, low production yields are typically obtained, motivating efforts to develop production processes that can overcome this bottleneck, enhance 2-PE yields and reduce the production costs. This review presents and discusses the latest advances in the bioproduction of 2-PE through microbial fermentation, in terms of producing strains, the optimization of cultivation processes, strategies to mitigate product toxicity, and the use of low value feedstocks. Novel applications for 2-PE are also highlighted.

## 1. Introduction

2-Phenylethanol (2-PE) is an aromatic alcohol with a rose-like scent, one of the most popular fragrances, being the major component of rose water. It is applied in a wide range of industries, such as in cosmetic, food and beverage industries. Due to its antimicrobial characteristics, it is used in laundry and home care products. It is generally recognized as safe (GRAS, 2858) and used in the pharmaceutical industry, mainly as a preservative, and as an additive in cigarettes [1,2,3,4,5].

**Figure 1:**
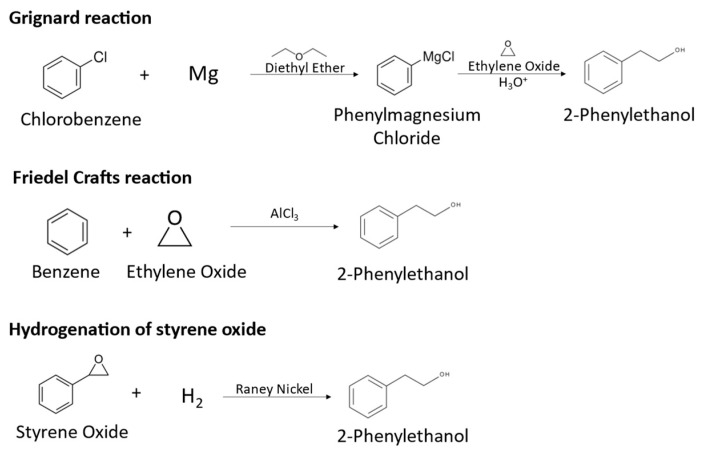
Synthetical routes for 2-PE production: Grignard reaction, Friedel–Crafts reaction and hydrogenation of styrene oxide.

The global market demand for 2-PE is increasing, reaching USD 255 million in 2021, and is expected to continue growing in the next years [1]. About 93% of the commercialized compound is obtained by chemical synthesis, from chlorobenzene, benzene or styrene oxide. For instance, the main synthetical routes for 2-PE production include Grignard and Friedel–Crafts reactions and the hydrogenation of styrene oxide. In Grignard reactions, chlorobenzene is converted to 2-phenylethanol in a series of reactions using diethylether as solvent, in which chlorobenzene is converted to phenylmagnesium chloride, which reacts with ethylene oxide at 100 °C to form phenylethoxy magnesium chloride, which is decomposed to 2-PE using sulfuric acid, as represented in Figure 1 [6]. This chemical route presents important disadvantages, such as the use of hazardous diethyl ether, the high temperatures needed and the low quality of the produced 2-PE, which is contaminated with reaction side products. Friedel–Crafts reactions consist in the alkylation of benzene with ethylene oxide using aluminum chloride at temperatures lower than 25 °C, producing 2-PE, as represented in Figure 1 [6,7]. However, the use of corrosive and environmentally hazardous reagents and the formation of side products affecting the quality of the product are major drawbacks of the process. The hydrogenation of styrene oxide uses Raney nickel as a catalyst, resulting in 2-PE, as represented in Figure 1. This process requires high pressure conditions due to the use of hydrogen gas and generates hazardous wastes. Overall, synthetic 2-PE production has serious drawbacks, namely the use of hazardous or corrosive chemicals, harsh conditions of temperature and pressure and strong acid environments and low selectivity. Additionally, the difficult separation and side products formation affect aroma quality and increase downstream costs [4,8,9]. Nevertheless, 2-PE is naturally present in a variety of plants such as roses, jasmine, buckwheat and tomato, being extracted mainly from rose petals. However, due to the complex and costly extraction process, the low concentration obtained from plants and weather-dependent production, its costs can be 250 to 300 times higher than the chemically synthesized compound and the production cannot meet the demand for the natural product [1,4,8]. In fact, for the obtention of the rose flavor, rose petals are usually manually harvested, and are subjected to a distillation process in the presence of water, obtaining an aqueous phase and a lipophilic phase. The aqueous phase corresponds to the rose water, with 2-PE being the main component, while the lipophilic phase is the essential oil, which also contains 2-PE in its composition [10].

The rising awareness among the consumers regarding environmental and health issues has increased the demand for natural products, leading to the pursuit and development of biotechnological alternatives for 2-PE production by the suppliers. In fact, the USA and European legislation recognize microbiologically produced flavors as natural, making biotechnological production an effective alternative [11,12]. Compared to the 2-PE extracted from plants, biotechnological processes have the advantage of not depending on weather conditions, plant diseases and trade restrictions, which strongly influence the price [13,14]. Moreover, these biotechnological processes use mild conditions and have high product selectivity and generate a lower amount of waste and less toxic wastes [14]. The price of biotechnologically produced compounds is still 10 to 100 times higher than the synthetically produced compound due to the low production yields and downstream separation costs. However, the use of cheap feedstocks, such as agro-industrial byproducts, can make the process economically attractive and simultaneously contribute to a circular economy [15,16].

This review explores the recent breakthroughs in the microbiological production of 2-PE, focusing on innovative strategies to boost productivity. Key topics include the application of in situ product removal (ISPR) techniques and the use of agro-industrial byproducts as cost-effective feedstocks. Additionally, this review highlights the unique properties of 2-PE and the range of applications, offering insights into how these advancements are shaping the future of 2-PE.

## 2. 2-PE Biotechnological Production

### 2.1. Metabolic Pathways

**Figure 2:**
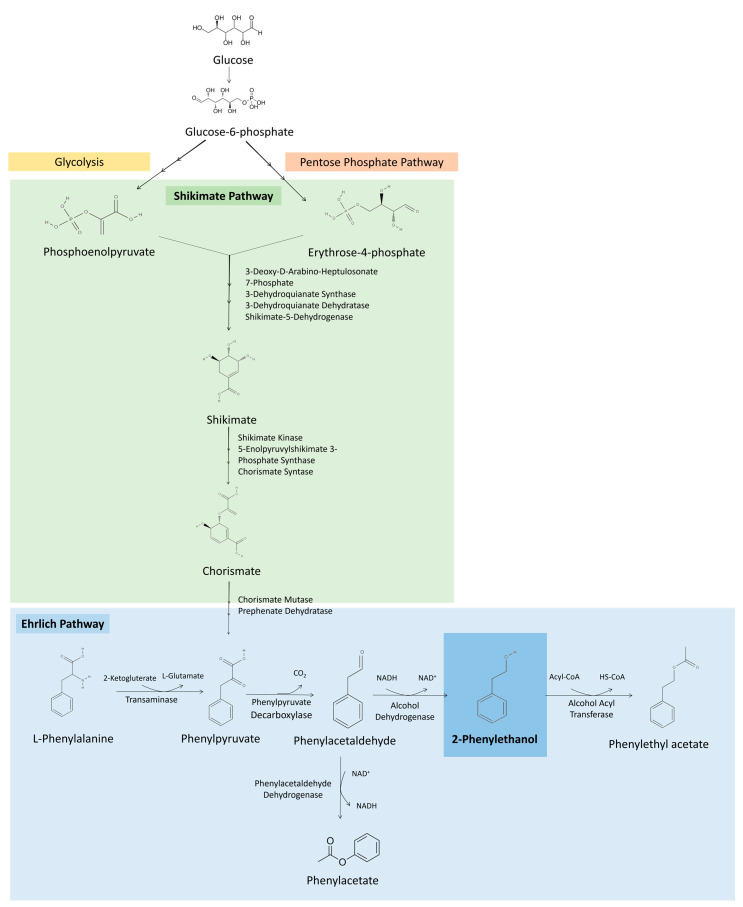
Metabolic pathways involved in 2-PE production. Ehrlich pathway for 2-PE production from L-Phe. Shikimate pathway for de novo 2-PE biosynthesis from erythrose-4-phosphate produced during glycolysis and phosphoenolpyruvate coming from the pentose phosphate pathway, resulting in phenylpyruvate that enters the Ehrlich pathway to be converted in 2-PE.

Several microorganisms, among bacteria, yeast and fungi, can produce 2-PE through different metabolic pathways. The main pathways for 2-PE production are the Ehrlich and Shikimate pathways. Among these, the Ehrlich pathway is considered the most efficient metabolic approach for 2-PE production [2,3]. Figure 2 illustrates the principal biosynthetic pathways involved in 2-PE production by microorganisms.

The Ehrlich pathway consists of the catabolism of amino acids (valine, leucine, isoleucine, methionine, phenylalanine, tyrosine and tryptophan) to the corresponding aromatic alcohols or acids. 2-PE production by this pathway is achieved in three steps from L-phenylalanine (L-Phe) (Figure 2). L-Phe is transaminated to phenylpyruvate, then decarboxylated, obtaining phenylacetaldehyde, and finally reduced to 2-PE, which can be further converted into phenylethyl acetate [4,17]. However, using *Saccharomyces cerevisiae* as a model, the initial transamination reaction is negatively affected by the presence of nitrogen sources that are not involved in the Ehrlich pathway, such as ammonia, proline and asparagine, being beneficial to have the amino acid as sole source of nitrogen in the broth to achieve higher 2-PE productions [17,18,19]. Additionally, it was found that when the culture is glucose limited, amino acids are converted mainly into aromatic acids, achieving a low concentration of aromatic alcohols [17].

Microorganisms can also produce 2-PE directly from glucose via the de novo shikimate (or also called phenylpyruvate) pathway (Figure 2). This pathway consists of condensation in successive steps of phosphoenolpyruvate obtained from glycolysis, and erytrose-4-phosphate from the pentose phosphate pathway. These compounds are converted into phenylpyruvate that enters the Ehrlich pathway to produce 2-PE. However, the availability of phosphoenolpyruvate and erytrose-4-phosphate for the Shikimate pathway is low, limiting the productivity of 2-PE production by this method [20]. The advantage of this metabolic pathway is the lower cost of glucose, compared with L-Phe [20,21]. Therefore, metabolic engineering strategies may be developed to enhance the activity of crucial rate-limiting enzymes, reduce feedback inhibition and consequently improve 2-PE production by de novo synthesis [20,21]. For example, using a multilevel metabolic engineering strategy, Zhan et al. redirected the carbon flux towards 2-PE production and eliminated the competing precursor pathways in *Bacillus licheniformis*, achieving 6.24 g/L of 2-PE from glucose, in a complex medium [22]. Similarly, by increasing phenylpyruvate availability using genetic engineering strategies in *Pichia pastoris*, Kong et al. were able to accumulate 1.15 g/L of 2-PE from glucose [23].

### 2.2. Producing Strains

Several microorganisms have the natural ability to produce 2-PE. The most relevant microorganisms reported for 2-PE production are yeasts. However, this capability can also be found in certain bacteria and fungi.

For instance, 2-PE production in the presence of L-Phe was reported in bacteria involved in cheese ripening *Brevibacterium linens*, *Proteus vulgaris*, *Proteus mirabilis* and *Psychrobacter*, as well as in *Mycobacteria* strains and in *Erwinia carotova* subsp. *atroseptica* [24,25,26,27,28]. Nonetheless, these bacteria are usually associated with low aroma production, generally below 152 mg/L. Interestingly, a newly isolated bacterium, isolated from river water from Angola, *Acinetobacter soli* ANG344B, was recently reported to produce 2-PE in high concentrations, not previously reported for wild-type bacteria, being able to produce 2.14 g/L from L-Phe, a value in the range of yeast production [29,30]. Metabolic engineering strategies are being applied to improve 2-PE production in bacterial strains, as detailed in Table 1 [21,22,31]. For instance, *Enterobacter* sp. CGMCC 5087 was found to produce 0.1 g/L of 2-PE through the Shikimate pathway using glucose as a carbon source and NH_4_Cl as a nitrogen source, and by the modification of that pathway it was possible to increase 2-PE production to 0.33 g/L [21]. Liu et al. reported a 2-PE production of 3.21 g/L by a modified *E. coli* strain with a new metabolic pathway identified in *Proteus mirabilis*, that originally could produce 0.08 g/L of 2-PE from L-Phe [31]. Likewise, Noda et al. reached a production of 2.5 g/L of 2-PE from glucose in 72 h in a modified *E. coli* strain from *Saccharomyces cerevisiae* [32]. The native *Bacillus licheniformis* could not produce 2-PE but possessed alcohol dehydrogenases that were capable of converting phenylacetaldehyde into 2-PE [22]. By different modifications of this strain, it was capable of producing 6.24 g/L of 2-PE from L-Phe and 6.43 g/L from glycerol and molasses [22,33,34].

Regarding fungi, *Annulohypoxylon stygium* strain S20 produced 2.33 g/L of 2-PE from L-Phe in 2 days, while *Aspergillus oryzae* RIB40 could produce 0.1 g/L of 2-PE, also from L-Phe, mainly though the Ehrlich pathway [35,36].

Yeast’s ability to produce 2-PE is widely recognized, being the most reported microorganism for biotechnological 2-PE production. *Saccharomyces cerevisiae* and *Kluyveromyces marxianus* are the most effective 2-PE producers, achieving 2-PE productions up to 4.5 and 1.45 g/L, respectively, as can be seen in Table 2 [37,38]. However, many other yeasts are also reported for 2-PE production, reaching similar 2-PE production levels. For example, *Yarrowia lipolytica*, *Wickerhamomyces anomalus* and *Pichia kudriavzevii* have been shown to produce up to 5 g/L of 2-PE, as summarized in Table 2 [23,39,40,41,42,43,44,45,46]. The ability of yeasts to produce 2-PE is frequently identified in wild-type strains screened from a variety of environments. For instance, *Wickerhamomyces anomalus* was identified from a screening of rice wine, while *Kluyveromyces marxianus* CCT7735 was isolated from a screening comprising different Brazilian environments such as diary industries and Amazonia rubber trees [45,47]. Further, 18 yeast species were identified as 2-PE producers in a screening involving plant material and spontaneously fermented food, and a recently isolated *Saccharomyces bayanus*, able to produce 2-PE in concentrations of 6.5 g/L in a fed-batch approach, was isolated from soy sauce mash [48,49]. Yet, strategies for genetically engineering yeasts to improve 2-PE production are also being investigated. For example, *Yarrowia lipolytica* underwent a metabolic engineering process to improve 2-PE by de novo biosynthesis, while *Kluyveromyces phaffi* was engineered to improve 2-PE production through the Ehrlich pathway and also to improve the tolerance towards the product [50,51].

Although several microorganisms are known for their ability to produce 2-PE, its production is not yet attractive for industrial applications due to the low titer and high production costs compared with the synthetic route. Strategies to improve 2-PE production go through the improvement of fermentation conditions, strain engineering and strategies to mitigate the toxic effect that 2-PE has on the producing microorganism. Additionally, the search for alternative and cost-effective substrates for 2-PE production will also improve the attractiveness of the biotechnological process.

### 2.3. Cultivation Process Optimization

The 2-PE production process is highly influenced by the cultivation medium composition and reactor operational conditions. These factors affect microbial growth and the activation of pathways leading to 2-PE production, which influences the process’s productivity and overall relevance [3,70]. Regarding the cultivation medium composition, nitrogen sources, nutrients and carbon sources are key factors for the 2-PE production process, while fermentation parameters such as pH, temperature and aeration also play an important role in the process.

#### 2.3.1. Influence of Nitrogen Sources

The presence of nitrogen sources in addition to L-Phe are reported to influence the activation of the Ehrlich pathway. In fact, L-Phe is reported to be a non-preferable nitrogen source, for yeasts as *Saccharomyces cerevisiae*, and in the presence of preferable sources of nitrogen, such as ammonium salts, the expression of some enzymes involved in the Ehrlich pathway are low or even negligible [40,71]. So, the use of L-Phe as the sole nitrogen source leads to an increase in 2-PE production, not only for *Saccharomyces cerevisiae*, but also for nonconventional yeasts such as *Wickerhamomyces anomalus*, *Candida* sp. and *Kluyveromyces marxianus* [72,73,74]. However, some studies have shown that the presence of moderate concentrations of yeast extract *in* the cultivation broth have a positive influence in 2-PE production since it is not only a source of nitrogen, but a source of important growth factors, vitamins and microelements needed for cellular metabolism [29,42,44,72]. Fan et al. reported an increase in 2-PE production from values under 2 g/L in the absence of yeast extract, to a 2-PE concentration of 2.77 g/L when 6 g/L of yeast extract was present in the cultivation broth of *Pichia kudriavzevii* [42]. Likewise, Bernardino et al. noted an increase in 2-PE production by the bacterium *Acinetobacter soli* ANG344B, from 0.87 g/L in absence of yeast extract to 1.77 g/L in the presence of 5 g/L of yeast extract [29].

#### 2.3.2. Influence of Carbon Sources

The carbon source is another key factor for cellular growth, L-Phe consumption and 2-PE production. L-Phe consumption depends on ATP or the electrochemical gradient generated from the metabolism of the carbon source, namely, glucose [46,47]. The most reported carbon source for 2-PE production is glucose and an increase in this carbon source concentration usually leads to increased 2-PE production until a certain level, that is related to the microorganism in study [42,45,47]. In yeasts, increased glucose concentrations usually lead to higher ethanol production, causing an inhibitory effect towards microorganisms and consequently affecting both L-Phe consumption and 2-PE production. Additionally, the synergistic toxic effect of ethanol and 2-PE further affects the process [47]. Different carbon sources such as glycerol, xylose, fructose and lactose were also explored for 2-PE production by yeasts, although the ability to use these substrates varies with the metabolism of each strain [46,52,68,69,72]. For instance, Braga et al. tested glucose and glycerol as carbon sources in different *Yarrowia* species. They found that despite glucose being the preferred carbon source for yeast growth, there was no statistically significant differences in the 2-PE production when glycerol was used by *Yarrowia lipolytica* CH 1/5, being able to produce 3.2 g/L of 2-PE [68]. *Meyerozyma* YLG18 was also cultivated using different carbon sources, including glucose, glycerol and xylose. Glucose resulted in the highest 2-PE production, of 1.25 g/L, while xylose led to a higher molar conversion from L-Phe. In contrast, glycerol resulted in the lowest 2-PE production and molar conversion, despite being expected to provide more NADH needed for the Ehrlich pathway [46]. A co-culture of *Kluyveromyces marxianus* and *Debaryomyces hansenii* was able to use lactose to produce 2-PE and ethanol, with ethanol serving as an alternative carbon source for both yeasts [52]. On the other hand, Gethins et al. found that *Kluyveromyces marxianus* produced low levels of 2-PE when lactose was used as the carbon source, in comparison with the use of glucose or fructose [72].

#### 2.3.3. Influence of Fermentation Parameters

Temperature and pH are also key factors for 2-PE production and culture growth, influencing the expression and activity of enzymes involved in the 2-PE production process. Often the optimal conditions for cell growth differ from those for 2-PE production [4,43,68,70]. For instance, *Pichia fermentans* L-5 was found to have the highest cellular growth at 25–30 °C, while the 2-PE production was maximized at 30–35 °C and pH 8.5. For lower pH values, 2-Phenylethyl acetate, a byproduct of the Ehrlich pathway (Figure 2), was not detected in broth, but at higher pH values, this aromatic compound was detected in different concentrations [43]. Mu et al. also reported different suitable conditions for cellular growth and 2-PE production, using a mixed microbial culture. In this case, low pH was favorable for cellular growth (pH 4.5), while pH 8 was more beneficial for 2-PE production with a temperature of 35 °C [70]. On the contrary, for *Yarrowia lipolytica,* a decrease in the pH from 7.5 to 5.5 improved 2-PE production, but different conditions of oxygen supplementation did not have significant differences in 2-PE productivity [68]. *Saccharomyces cerevisiae* preferred a temperature for cellular growth (25 °C) that was not coincident with the preferred temperature for aroma production (35 °C). The oxygen requirements for these two metabolic processes differ; an aeration rate of 1.3 vvm is optimal for yeast growth, while 2 vvm favors aroma production [53,75]. Thus, the conditions that assure the best 2-PE production process are dependent on the microorganism. Furthermore, the production of 2-PE is also affected by the toxicity of the product itself.

## 3. Cytotoxicity of 2-PE

One of the most limiting factors for 2-PE production is its cytotoxicity to the producing organism, similar to what is observed in the production of other microbial aromas, such as vanillin and benzaldehyde [76,77]. The amphipathic nature of 2-PE interferes with the cell membrane, decreasing the lipid order and increasing membrane fluidity by disrupting the phospholipid components, acting as a bacteriostatic agent [78,79]. This interaction disturbs nutrient and ion transport, energy generation and cellular communication, ultimately affecting cellular homeostasis, leading to growth inhibition and even cell death [78,80,81,82,83].

The inhibitory mechanism of 2-PE for *Saccharomyces cerevisiae* was studied at the molecular level, revealing an inhibition of nitrogen and ion uptake, and feedback repression in the synthesis of alcohol dehydrogenase, which is a crucial enzyme in the Ehrlich pathway, resulting in 2-PE production limitation. In the presence of 2-PE, yeasts increase mitochondrial activity and initiate differentiation by spore formation instead of budding, indicating a lack in yeast nutrition [84]. *Kluyveromyces marxianus* was not able to grow or consume glucose after an exposure to 3 g/L of 2-PE, and an increase in membrane permeability was noticed, accompanied by changes in cell membrane composition to counteract the damages caused by 2-PE [85].

Regarding bacteria, the response of *Bacillus licheniformis* to the contact with 2-PE was investigated [83]. This strain is a 2-PE-tolerant strain that can tolerate up to 5 g/L of the aroma [33]. The 2-PE contact with cells induces reactive oxygen species (ROS) formation, causing damage to DNA, RNA, ribosomes, the cell wall and metabolism. The cell morphology is also changed under 2-PE stress, with increased width. This stress leads to cellular responses, including protein synthesis or the stimulation of the tricarboxylic acid (TCA) cycle to increase the supply of NAD+ and NADP+, to deal with oxidative stress. The distribution of the head groups of phospholipids is also changed to avoid the access of 2-PE to the cells. The contact of the aroma with this bacterial strain also induces the downregulation of transporters to reduce energy consumption, meeting the increased energy needs for 2-PE resistance [83].

In fact, 2-PE-producing microorganisms are inhibited by the product in concentrations between 2 and 5 g/L, depending on the species. For instance, *Saccharomyces cerevisiae* tolerates 2-PE concentrations below 4 g/L, with the cellular growth completely inhibited at that concentration, while no growth inhibition was observed for concentrations below 0.6 g/L [54,59,82]. On the other hand, *Kluyveromyces marxianus* has a lower tolerance to 2-PE, being able to produce the aroma compound in concentrations below 2 g/L, while in the presence of 3 g/L of exogenous 2-PE, a decrease in the specific growth rate by 80% was observed [38,41,85]. Regarding bacteria, *Bacillus licheniformis* DW2 was reported to tolerate 5 g/L of 2-PE, having a long lag period (16 h) in these conditions, with the growth being completely impaired in the presence of 6 g/L of the aroma compound [33]. *Acinetobacter soli* ANG344B was reported to be negatively affected by the presence of 1 g/L of 2-PE and beyond 4 g/L of the aroma compound, bacterial growth is completely inhibited [29].

To improve 2-PE production, strategies to overcome product toxicity are being developed. These strategies include genetic modifications of the microorganisms to increase their tolerance towards the desired product and finding alternatives to remove the product from the cultivation broth, while it is being produced, allowing the microorganism to continue 2-PE production [4,86,87].

## 4. In Situ Product Removal and Product Recovery

The cytotoxicity of 2-PE towards the producing microorganism is a bottleneck for flavor production, limiting the viability of the process and therefore its implementation at an industrial scale due to the low titer and productivity. To improve the 2-PE production process, in situ product removal (ISPR) techniques have been extensively studied. These processes consist of removing the product that causes inhibition from the fermentation broth as soon as it is produced. This strategy avoids the exposure of the producing microorganism to high concentrations of the product and consequently allows continuous production, usually at maximal production rates and for longer periods, resulting in higher productivities [88,89,90]. The use of ISPR techniques can also minimize product loss by degradation or uncontrolled evaporation and facilitate the downstream processes, thus improving process feasibility [88,89,90].

To increase 2-PE production several approaches based on the use of ISPR techniques have been studied, such liquid–liquid extraction and adsorption, membrane-based processes and supercritical fluids [3].

### 4.1. Liquid–Liquid Extraction

Liquid–liquid extraction is one of the most reported methods and is based on the difference in the solubility of the target product in two immiscible liquids [90]. The process involves an aqueous phase, where the biotransformation occurs and an organic phase with high affinity for 2-PE, where the aroma is retained. The selection of the solvents is crucial for the process. To be considered a suitable extractant, the solvent must have specific characteristics. The solvent cannot be toxic to the microorganism or reactive with other components of the cultivation media and cannot be available for use as a carbon source by the microorganism. It has to be chemically and thermally stable, non-flammable, of low viscosity and inexpensive. The solvent has to be water immiscible and provide a good phase separation from the aqueous phase and the target product should have a large partition coefficient in the solvent. The product should be recoverable from the solvent and, regarding the production of flavor compounds, the solvent must be approved for use in food processing [88,90,91]. However, hydrophobic solvents can interact with the membrane of the microorganism and may have a harmful effect, but these limitations vary with the microorganisms and their membranes’ characteristics [88].

The use of liquid–liquid extraction for 2-PE production has been reported to improve the aroma production process, up to 10 g/L, as it can be seen in Table 2, using solvents such as oleic acid, rapeseed oil, fatty acid methyl ester (FAME), polypropylene glycol, biodiesel, ionic liquids (ILs) and ethyl acetate [15,41,46,55,90,91,92,93]. However, some limitations were identified using these solvents; for instance, the presence of oleic acid and FAME were reported to have a negative influence on cellular growth and viability [46,62,91]. The formation of stable emulsions during bioreactor cultivation in the presence of oleic acid, hindering phase separation and the on-line monitoring of biomass, was also described [91]. Even though ILs are commonly reported to be green solvents with unique solubility properties and high stability, their high costs and the potential risk of toxicity during the extractive fermentation are still disadvantages to use these solvents, so they are under continuous investigation [90].

### 4.2. Adsorption

The adsorption of the product of interest is also reported using a solid material that has high affinity for the product. These materials are non-volatile, non-biodegradable and low cost. The use of these adsorbents has advantages over organic solvents, such as not affecting the product’s organoleptic properties or being toxic to the producing microorganisms [3,4]. This is a mild technique able to absorb products with both high and low molecular mass. This technique can be performed in columns through which the fermentation broth can be kept in contact until the full capacity of the material to adsorb the product is achieved [90]. However, the capacity of the adsorbents for the target product might decrease due to nonspecific interactions with cells, cellular debris, medium components and byproducts [88,90].

Several polymeric adsorbents have been reported to enhance the 2-PE production process by coupling the product adsorption to the cultivation, as can be seen in Table 2. To be a suitable adsorbent for 2-PE, the polymer should be able to interact with the functional groups of 2-PE, the aromatic ring and/or the hydroxyl group by π or hydrogen interactions, respectively [38]. The crystallinity degree is also an important characteristic for product adsorption, since the polymeric material should have space to accommodate the product, and this can be achieved by having an amorphous structure [38].

Non-polar polymeric adsorbents, such as the macroporous resins D101 (from Chemical Plant of Nankai University), HZ818, FD0816 (both from Shanghai Huazhen Science & Technology Co., Ltd., Shanghai, China.) and Amberlite XAD 4 (from Thermo Scientific, Waltham, MA, USA), with a cross-linked polystyrene structure that allows the interaction with the product through the aromatic ring, have been used to improve 2-PE production by different *Saccharomyces cerevisiae* strains and the bacterium *Acinetobacter soli* ANG344B [62,63,64,84,85], as described in Table 2. Even though these polymers are able to interact with both 2-PE and L-Phe, they have more affinity for the aroma than the precursor. The block copolymer resins Hytrel G3548 and Hytrel 8206 (from DuPont, Wilmington, Delaware, USA), composed of polybutylene ester and polyether, are able to interact with the hydroxyl group of 2-PE by hydrogen interactions, and were evaluated to improve the aroma production by *Saccharomyces cerevisiae* and *Kluyveromyces marxianus*, respectively [38,53]. The use of these polymers was reported in batch cultivation experiments and the amount of polymer used varied between 2 and 17% *w*/*v*, allowing the production of 4 to 14 g/L of 2-PE. Increasing the amount of resin in contact with the cultivation broth also increases the aroma production, so semicontinuous processes have been attempted, applying the resin in filter cloth bags inside the bioreactor or in columns through which the cultivation broth passes [38,94,95]. For instance, Wang et al. increased the production of 2-PE by *Saccharomyces cerevisiae* R-UV3 using the non-polar macroporous resin FD0816, from 4.38 g/L in a fed-batch fermentation process to 13.7 g/L when using 132 g of dry resin in a 3 L bioreactor [94,95]. By replacing the resin and making the process semicontinuous, the 2-PE production increased to 32.5 g/L with a production rate of 0.54 g/L·h. Passing the cultivation broth through the columns filled with resin, the production rate could even increase to 0.9 g/L·h, with the fouling of the membrane used to separate the biomass being the limiting step. Likewise, Gao and Daugulis could improve the aroma production by *K. marxianus*, from 1.45 g/L of 2-PE in a single-phase batch run, to 13.7 g/L using 500 g of Hytrel 8206 [38]. Further, in a 3 L bioreactor cultivation operating in a fed-batch mode, the increase in resin to 900 g led to a 2-PE production of 20.4 g/L, with an overall productivity of 0.43 g/L·h. Using this strategy, only 1.4 g/L of 2-PE were present in the aqueous phase, avoiding product toxicity.

Different strategies for in situ product adsorption were also investigated, namely the entrapment of an organic solvent, dibutylsebacate, into a polymeric matrix of polyethylene or alginate to form a stable resin adsorbent, as well as the use of a polydimethylsiloxane sponge or hydrophobic polymethylmetacrylate microspheres [60,75,96,97].

### 4.3. Membrane-Based Extraction

Membrane-based processes for 2-PE separation are also being investigated and membrane-based solvent extraction is the most reported process, as it is summarized in Table 2, allowing 2-PE production up to 21 g/L [57,58,98]. This approach is based on a liquid–liquid extraction process where an organic solvent, that acts as an extractant, passes through a hollow fiber membrane module and contacts the cultivation media, with the product continuously extracted for the organic phase [57,98,99,100]. These hollow fiber contactors can be advantageous by increasing the contact area with the solvent and avoiding the formation of emulsions with the aqueous media. Nevertheless, the membrane pores can be clogged with the biomass produced in the cultivation process, decreasing the efficiency of the process [4,90,99]. These approaches can be applied as submerged membranes in the cultivation broth (pertraction) or as separated modules in an external loop.

Organophilic pervaporation and supercritical CO_2_ were also reported for continuous 2-PE extraction from the production fermenter, but with limited improvement in aroma production due to the high temperatures needed to favor the process’s driving force and the toxicity of the CO_2_ to the microorganisms, respectively [99,101].

Alternatives using combinations of these approaches are also being investigated to overcome the toxicity of 2-PE that limits further aroma production, for instance, the combination of liquid–liquid extraction and adsorption [59,61,93].

The processes for ISPR described above showed improvements in 2-PE production, but there are several ways to do this. The choice of an extraction method to connect to the fermentation process should be based on the product’s physical, chemical and biochemical characteristics, such as the molecular mass, volatility, solubility, charge and hydrophobicity. Moreover, it should meet the requirements of purity, according to the applications [90]. Due to the low molecular weight of 2-PE and hydrophobicity, ISPR techniques such as gas stripping, solvent extraction, pertraction, vacuum distillation, adsorption, solvent extraction, the use of supercritical fluids, evaporation, permeation and immobilization are techniques suitable to be considered in ISPR processes for 2-PE extraction [90].

## 5. Alternative Substrates to Produce 2-PE

Agro-industrial wastes are known to be a source of minerals, proteins and sugars with a growing potential to be used in circular economy-based approaches [102]. The use of these low value nutrition-rich materials to produce value-added compounds, contributes to promoting a sustainable management of the residues, reducing waste disposal to the environment and decreasing production costs, due to their high availability and low price [3,102]. However, these raw materials have a variable composition and often need pretreatments to be used [3]. The use of agro-industrial wastes as carbon sources in 2-PE production processes is being investigated in submerged fermentation and solid-state fermentation technologies. Examples are summarized in Table 2.

Different wastes and byproducts were reported to be used as carbon sources for 2-PE production by yeasts, in submerged fermentation systems (Table 2). Some of these residues need hydrolysis processes to release fermentable sugars from the complex polymers. For instance, corn stover, rich in glucose and xylose, was hydrolyzed in alkaline conditions, while forestry residues, such as the bark of pine wood, rich in glucose, had to pass through acid hydrolysis, and both were supplemented with L-Phe to allow 2-PE production [67,69]. Grape must, rich in glucose and fructose, whey, rich in lactose and proteins, and crude glycerol were also used as part of the cultivation media of different yeasts, in the presence of L-Phe [15,52,65,66,68]. These media supported the production of 0.77 to 6.37 g/L of 2-PE as presented in Table 2, considering wild-type organisms.

Some residues are also being investigated to produce 2-PE by de novo synthesis, in the absence of L-Phe, using tequila vinasses and cheese whey. Nevertheless, the production by this metabolic pathway leads to lower 2-PE production (0.065 and 0.96 g/L), compared with the Ehrlich pathway for wild-type microorganisms [73,103]. However, metabolically engineered microorganisms are also being investigated to produce 2-PE from low value feedstocks; for example, *Bacillus licheniformis* can produce 6.43 g/L of 2-PE from a combination of molasses and crude glycerol [34].

The aforementioned processes are based on submerged fermentation systems, but solid-state fermentation strategies are also being studied, using sugar cane bagasse or red apple pomace supplemented with L-Phe, resulting in 20.1 or 25 mg of 2-PE per gram of used waste [16,104].

Despite the promising results obtained with these low value wastes and byproducts, to the best of my knowledge, the use of a feedstock that could work simultaneously as a carbon and L-Phe source was not reported. In addition to the low L-Phe content in the wastes, the presence of other nitrogen sources, such as different amino acids, influences the Ehrlich pathway efficiency for 2-PE production [16,52]. Thus, media supplementation with L-Phe is an essential step for an efficient and high titer 2-PE production process.

Economic projections of 2-PE production using wastes, namely whey, have been investigated in order to access the viability of the implementation of such a process. For instance, Conde-Báez et al. studied two economic scenarios involving 2-PE production by *Kluyveromyces marxianus* using whey as a carbon source and L-Phe as a precursor, based on a production of 0.78 g/L of 2-PE: the first one in which 2-PE is sold as an additive and the second one in which the compound is sold as a high purity product [105]. The first scenario consists of a pasteurization of whey and further fermentation process for 2-PE production. In this case, operational costs were estimated and fixed costs were projected leading to a final price of the product of USD 207.05 by tone. The second scenario adds the separation and purification steps, with an estimated final price of USD 1000 per kg. However, this second projection needs more detailed studies on separation and purification processes to have a more accurate projection [105]. On the other hand, Puga-Córdova et al. reported an economic projection in which the estimated total production cost of 2-PE from whey is USD 144.46 per kg, including a liquid–liquid extraction column and two distillation columns for product purification and solvent recovery [106]. Considering that the market values of naturally extracted rose water can reach USD 70 per L and rose essential oil can cost USD 7500 per kg, these approaches for 2-PE production using wastes, in addition to the advantage of a circular economy, are also cost-effective, resulting in a production process with economical potential [105,106,107]. Yet, few reports have been published regarding this subject, and further investigation into 2-PE production would benefit from this economical approach.

## 6. 2-PE Properties and Possible Applications

Even though 2-PE is being used in various fields, more applications are currently being investigated, mainly in health and food areas. Studies showed that 2-PE can be used as an odorant in olfactory training to improve the olfactory function in patients with post-infectious olfactory dysfunction or a loss of smell, caused by respiratory tract viruses [108]. The aroma is also reported to have sedative effects when administered by inhalation to mice, causing a significant decrease in the amount of spontaneous motor activity in a dose-dependent manner [109]. Other studies in mice showed that 2-PE can have anti-depressive effects, by acting on the central nervous system, suggesting that the inhalation of rose oil, which is rich in 2-PE, may be effective against depression and stress-related diseases [110]. Similarly, after corticosterone treatment to induce an anxio-depressive-like phenotype in female mice, a prolonged 2-PE inhalation allowed the regulation of the activity of neural circuits involved in sensory, emotional and feeding behaviors [111].

Considering the antimicrobial properties, 2-PE can increase antifungal effect when used in combination with Fluconazole and Itraconazole, having the potential to be applied in chronic and recurrent candidal vulvovaginitis in animal models [112]. Rowley et al. evaluated 2-PE usage in biomaterials, namely polyglobalide-based organogels that showed antimicrobial activity against *Staphylococcus aureus* and *Escherichia coli* [113]. Together with its rheology modifier properties, these materials are promising candidates to be employed in personal care products for the prolonged release of fragrant molecules and as antimicrobial gels. Zhu et al. found that 2-PE has antityrosinase and antimicrobial activities towards *E. coli* and *Ralstonia solanacearum.* These bacteria are known to cause disease in various food crops such as tomato, banana and ginger, and the enzyme tyrosinase that causes browning in fruits and vegetables can be reversibly inhibited by the aroma molecule [114]. Still related to its antimicrobial activity, 2-PE could suppress *Botrytis cinerea* growth in vitro, in strawberries and in postharvest tomatoes, reducing the disease without affecting the fruit quality, in a dose-dependent manner, by inducing reactive oxygen species stress and cell membrane disruption, making it a promising natural alternative to control gray mold in strawberries and tomatoes [115,116]. Additionally, this aroma compound could inhibit *Phytophtora infestans*, a pathogen causing the rot of potato tubers, by blocking the oxidative phosphorylation pathway, leading to the reduction of ATP levels and consequently cell death [117]. 2-PE was also investigated to protect citrus from infection with *Penicillium* molds and it could inhibit blue and green mold in vitro and in vivo, without affecting the product quality [118].

## 7. Future Perspectives

The future of biotechnological 2-phenylethanol (2-PE) production is poised at the intersection of scientific innovation, economic feasibility and environmental sustainability. The shift from traditional petrochemical methods to biotechnological processes represents a broader trend towards greener and more sustainable industrial practices. However, realizing the full potential of biotechnological 2-PE production will require overcoming significant technical and economic challenges. The key to its success lies in improving economic viability through enhanced yields, reduced production costs, and addressing the regulatory and consumer acceptance issues. Continued research and innovation are essential to overcome these barriers and fully realize the potential of biotechnological 2-PE production. The progress will depend on continued investment in research and development, as well as on the ability of the industry to adapt to changing market demands and regulatory landscapes.

The improvement of microbial 2-PE production processes to become economically and industrially attractive is being investigated through different approaches encompassing techniques such as the discovery of more productive species, the application of metabolic engineering techniques, synthetic biology and the development of improved and cost-efficient production processes. This is being attempted by using techniques to avoid product toxicity, such as ISPR approaches, and the use of low-cost feedstocks, to reduce the production costs and make the process more environmentally friendly. These approaches are developing steadily but it is essential to evaluate the costs of these technologies and scale-up the processes.

Ultimately, the success of biotechnological 2-PE production will hinge on its ability to deliver not just on sustainability, but also on economic efficiency and product quality, ensuring that it meets the needs of both producers and consumers in a rapidly evolving global market.

## Figures and Tables

**Table 1:** Engineering strategies applied in bacteria to improve 2-PE production.

Strain	Engineering Strategy	Improved Production (g/L)	Reference
***Enterobacter *** **sp. CGMCC 5087**	Shikimate pathway: transformation with two genes encoding rate-limiting enzymes from *Escherichia coli* and overexpression.	0.33	[21]
***E. coli/Proteus mirabilis *** **JN458**	Mimic the new *P. mirabilis* pathway in *E. coli* and construction of a cofactor regeneration system.	3.21	[31]
***E. coli/Saccharomyces cerevisiae***	Shikimate pathway: introduction of a modified phenylpyruvate decarboxylase gene from *S. cerevisiae* in *E. coli.*	2.5	[32]
***Bacillus licheniformis *** **DW2**	Ehrlich pathway: introduction of Ehrlich pathway and blocking of competing pathways to redirect the carbon flux to that pathway.	6.24	[22,33]
***Bacillus licheniformis***	Shikimate pathway: transformation to replace sucrose metabolism and increase phosphoenolpyruvate availability in the cell.	6.43	[34]

**Table 2:** Overview of 2-PE wild-type production strains, production method and production parameters. Y(P/S) represents the 2-PE production yield from L-Phe and Rp represents the volumetric productivity.

Strain	Method	Carbon Source	L-Phe (g/L)	2-PE (g/L)	Y(P/S) (g/g)	Rp (g/L·h)	Reference
***Acinetobacter soli *** **ANG344B**	Batch 2 L bioreactor	Glucose	5	2.14	0.45	0.10	[29]
***Wickerhamomyces anomalus *** **1D6**	Single dose fed-batch 5 L Bioreactor	Glucose	5	4.73	-	0.10	[45]
***Yarrowia lipolytica *** **NCYC3825**	Batch Shake flask	Glucose	7	1.98	0.31	0.02	[39]
***Kluyveromyces marxianus *** **and *Debaryomyces hansenii* (co-culture)**	Batch Shake flask	Lactose	4	2.55	0.89	0.03	[52]
***Zygosaccgaromyces rouxii *** **M2013310**	Batch Shake flask	Glucose	-	3.58	-	0.05	[40]
***Saccharomyces cerevisiae *** **Ye9-612**	Fed-batch 3 L Bioreactor	Glucose	10	4.5	0.82	0.065	[37]
***Saccharomyces cerevisiae *** **BCRC 21812**	Batch 250 mL Bioreactor	Glucose	4	2.53	0.69	0.013	[53]
***Pichia kudriavzevii *** **YF1702**	Batch Shake flask	Glucose	10.7	5.09	-	-	[42]
***Candida Glycerinogenes *** **WL2002-5**	Batch 5 L Bioreactor	Glucose	7	5	0.71	0.11	[44]
***Saccharomyces cerevisiae *** **JM2014**	Batch 6.2 L Bioreactor	Glucose	6	3.60	-	0.05	[54]
***Saccharomyces bayanus *** **L1**	Fed-batch 5 L Bioreactor	Glucose	9.7	6.5	0.67	0.108	[49]
***Kluyveromyces marxianus *** **CBS 600**	Fed-batch ISPR PPG 1200 2.4 L Bioreactor	Glucose	7	10.2	0.61	0.33	[55]
***Saccharomyces cerevisiae *** **JM2014**	Batch ISPR rapeseed oil 4.5 L Bioreactor	Glucose	5	9.79	-	0.14	[15]
***Saccharomyces cerevisiae *** **AM1-d**	Batch ISPR [HMPyr][NTf2] Shake flask	Glucose, saccharose	6	16.46	-	0.34	[56]
***Kluyveromyces marxianus *** **WUT240W**	Continuous ISPR Ethyl acetate 4.8 L Bioreactor	Whey	5	1.15	1.12	0.057	[41]
***Kluyveromyces marxianus *** **ITD0090**	Batch ISPR Oleyl alcohol membrane contactor 5 L Bioreactor	Glucose	8	3.02	-	0.054	[57]
***Kluyveromyces marxianus *** **CBS 600**	Batch ISPR hollow fiber, Miglyol 2 L Bioreactor	Glucose	9	4.0	-	0.29	[58]
***Saccharomyces cerevisiae***	Fed-batch ISPR pertraction–adsorption, octane Bioreactor	Glucose	11 × 6.5 g	2.69	0.65	0.498	[59]
***Saccharomyces cerevisiae *** **Ye9-612**	Fed-batch ISPR microspheres 3 L Bioreactor	Glucose	10	7.05	-	-	[60]
***Saccharomyces cerevisiae***	Fed-batch ISPR hollow fiber + adsorption Macronet MN270 15 L Airlift bioreactor	Glucose	45 g	11	-	0.15	[61]
***Kluyveromyces marxianus *** **CBS 600**	ISPR pervaporationBioreactor	Molasses + glucose	9	2.20	0.34	-	[62]
***Acinetobacter soli *** **ANG344B**	Batch 2 L Bioreactor ISPR adsorption Amberlite XAD 4	Glucose	12	6.99	0.58	0.17	[62]
***Kluyveromyces marxianus***	Semi-continuousISPR adsorption Hytrel 8206 resin 3 L Bioreactor	Glucose	26+	20.4	0.67	0.43	[38]
***Saccharomyces cerevisiae *** **BD**	Batch ISPR adsorption D101 resin Shake flask	Sucrose	12	6.17	0.51	0.23	[63]
***Saccharomyces cerevisiae *** **P-3**	Batch ISPR adsorption HZ818 resin Shake flask	Molasses	12	6.6	0.55	-	[64]
***Kluyveromyces marxianus***	Batch 24-well plates	Sweet whey	4.5	1.2	-	0.025	[65]
***Kluyveromyces marxianus *** **CBS 6556**	Batch 2 L Bioreactor	Grape must	3	0.77	0.62	0.0077	[66]
***Kluyveromyces marxianus *** **NRRL Y-1109**	Batch Shake flask	Forestry residues	4	1.09	-	-	[67]
***Yarrowia lipolytica *** **CH 1/5**	Fed-batch Bioreactor	Crude glycerol	8 + 4	3.2	0.29	0.0143	[68]
***Pichia fermentans *** **WUT36**	Batch Shake flask	Corn stover	5	3.67	-	0.05	[69]

## Data Availability

Not applicable.
